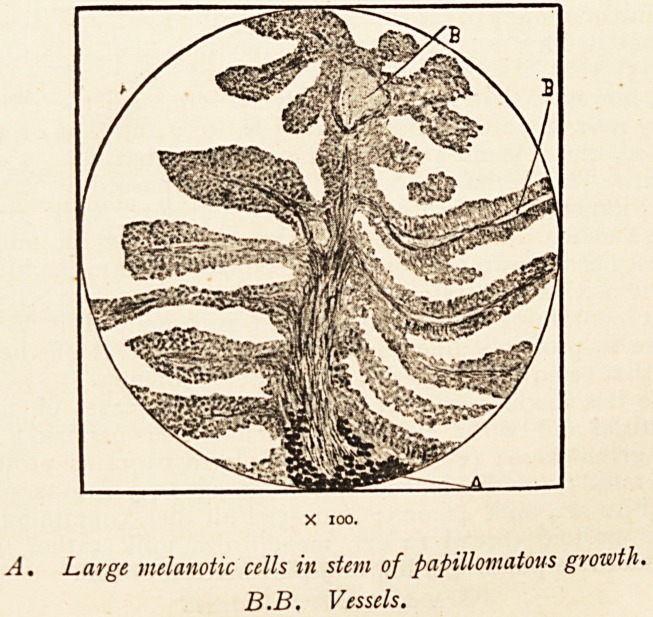# So-Called "Duct Cancer" of the Breast, with the Account of a Case of Large Recurrent Duct Papilloma

**Published:** 1894-03

**Authors:** Charles A. Morton

**Affiliations:** Surgeon and Surgical Pathologist to the Bristol General Hospital; Pathologist to the Hospital for Children and Women; Demonstrator of Anatomy, University College, Bristol


### SO-CALLED "DUCT CANCER" OF THE BREAST, WITH THE ACCOUNT OF A CASE OF LARGE RECURRENT DUCT PAPILLOMA.

BY

CHARLES A. MORTON, F.R.C.S. Eng.,

*Surgeon and Surgical Pathologist to the Bristol General Hospital; Pathologist to the Hospital for Children and Women; Demonstrator of Anatomy, University College, Bristol.*

---

MANY papillomatous cystic tumours of the breast have been described as "Duct Cancers."[1] Mr. Roger Williams[2] has pointed out that this is inaccurate, but I would suggest that

[1] Barker, *Brit. M. J.*, 1890, vol. i, p. 590. Pollard, *Tr. Path. Soc.*, 1886, p. 483. Pitts, *Ibid.*, 1888, p. 320. Battle, *Ibid.*, 1888, p. 322. Robinson, *Ibid.*, 1890, p. 221. Bowlby, *St. Bart. Hosp. Rep.*, 1888, p. 263, and *Lancet*, 1893, vol. i., p. 1369. Masterman, *St. Bart. Hosp. Rep.*, 1891, p. 193. Robinson, *Tr. Path. Soc.*, 1889, p. 285, and 1891, p. 229. [2] *Lancet*, 1892, vol. i., p. 858.

certain features which are still held to justify the name "duct cancer" as applied to these growths do not furnish sufficient ground for so calling them.

We find a cyst of the breast packed tightly with papillomatous growth, and therefore presenting the appearance of a solid tumour; and moreover, on microscopic examination we find a condition closely resembling the alveoli of cancer, as when the branching processes are cut across spaces are seen filled with (or lined by) epithelium; and in some of these tumours the papillomatous processes are described as joining and forming trabeculæ. The resemblance in some sections to cancer is a very close one, but we can generally in some parts define the arborescent papillomatous growth.

It is maintained that these tumours belong to the carcinomas because they grow from the epithelium of the ducts and infiltrate the parts around. This infiltration is said to be alone sufficient for classing them amongst the malignant tumours and distinguishing them from the simple papillomas. But is this infiltration of the surrounding fat alone sufficient for classing them as malignant tumours? The papillomatous growths in an innocent ovarian cyst may quickly burst through the cyst wall, and if that cyst was surrounded by tissue as it is in the breast, probably the extruding papillomatous growth [illegible] invade it; but the tumour is not therefore a cancer, though of course some of these papillomatous ovarian cysts are, but not necessarily every cyst with extruding papillomatous growth. And papillomatous masses from ovarian cysts have been found growing into the interior of the uterus.[1] Here the cyst could hardly have simply ruptured through the thick wall of the uterus and thus allowed the papillomatous growths to protrude, almost certainly the papillomatous growth mu[illegible] have itself penetrated the wall of the uterus.

That cysts with papillomatous growth were found in the fat around the breast in one or two of Mr. Bowlby's cases does not seem to me any argument in favour of regarding the disease as cancer, seeing how small outlying masses of gland tissue are found in the fat around the breast in which cystic change might

[1] *Johns Hopkins Hosp. Rep.*, vol. iii., 18[illegible]3, p. 28.

readily occur, for these tumours are not always found only in the neighbourhood of the larger ducts.

Are these cases then clinically malignant? There are a few cases on record which show that they may recur locally, such as Butlin's celebrated case of multiple recurrences,[1] and Bowlby's recorded in *St. Bartholomew's Hospital Reports*, 1888. Robinson's[2] growth has also recurred; it is described as a cyst, but not as if made up of cysts with intracystic growth. To these cases I must add the one which I now publish. From the description of Godlee's case,[3] which is the only one on record in which the glands were affected, I cannot make out the nature of the growth, but Pollard[4] says it resembled his cases, which were certainly only duct papilloma. The nature also of Shattock's rib specimen[5] seems uncertain; the description of it does not read like that of duct papilloma.

The malignancy then of these growths is only shown by their local recurrence, *i.e.*, they are no more malignant than a small spindle celled sarcoma or "recurrent fibroid," and in the great majority of cases they never recur at all. Why, then, should we call them cancers? Certainly the term cancer now means pathologically nothing more than that the growth is epithelial in origin; but clinically it expresses in many cases a malignant tendency, varying of course with the locality of the growth. A cancer of the breast is at any rate a very malignant form, and are we therefore justified in calling these cystic papillomas cancers, even if some of them recur locally? I think not. I should even object to call them malignant tumours, inasmuch as there are all degrees of malignancy, and to speak of a malignant tumour conveys no definite meaning. It may imply local recurrence at long intervals, as in the case of a recurrent fibroid, or gland infection as in epithelioma of the lip, or the most terribly fatal metastatic growths in internal organs combined with the other two. If, then, we speak of a cystic papillomatous tumour of the breast as malignant, we ought to be most careful in what sense we use the term.

I have carefully referred to all the cases Mr. Roger Williams

[1] *Tr. Path. Soc.*, 1887, p. 343. [2] *Ibid.*, 1891, p. 229. [3] *Ibid.*, 1876, p. 270. [4] *Ibid.*, 1886, p. 483. [5] *Ibid.*, 1888, p. 324.

mentions[1] as examples of tubular cancers, published in this country, and nearly all seem to me instances of duct papilloma, and to many of them I have already referred as such. Robinson[2] also quotes many of these cases in support of the description of his first variety of duct cancer—cases which are, I think, cystic papillomas.

### CASE OF RECURRENT DUCT PAPILLOMA.

A woman, 53 years of age, was admitted into the Bristol General Hospital in November, 1892, with a large recurrent tumour of the breast. She was under the care of Mr. Pickering, and I am indebted to him for permission to publish the case, in which I as registrar took much interest, and which I investigated pathologically. In the museum of the Hospital I discovered the primary growth, and I have made microscopic sections of it. I propose first to describe the growth, and then the recurrence. It was removed by Mr. Pickering three years ago. Almost certainly the whole breast was removed, as the nipple had disappeared when she was readmitted with the recurrence. The growth is a lobulated round mass, composed of a number of cysts filled with friable closely packed tissue. These cysts form the bosses on the surface. Between the cysts in the interior are some tracts of fibrous tissue. The whole growth at first sight appears solid, but on careful inspection it is seen to be made up of these cysts with tightly packed intracystic growth. The tumour measures 4½ by 3 in. A portion of one cyst-wall and the intracystic growth adjoining was removed, and, after hardening in strong spirit, embedded in celloidin and then cut. The embedding in celloidin was necessary, as the intracystic growth was so friable. The cyst-wall is in parts fibrous, but the larger area of the section is infiltrated with small round cells, which stain deeply with eosine, but no part of them takes the logwood stain. They do not, however, look like extravasated blood. In parts there are pigmented cells like a melanotic sarcoma. The intracystic growth is seen to be composed of papillomatous processes of epithelial cells with well-stained nuclei (with logwood), cut in all directions, with fibrous stems between them, here and there containing one or more vessels.

[1] *Lancet*, 1892, vol. i., p. 858. [2] *Ibid.*, 1892, vol. ii., p. 73.

The growth began to recur two years after removal. It gave rise to no pain. When she was admitted there was, under the old scar, a hard, irregular mass, the size of a cocoa-nut, hanging down by its weight towards the axilla. It did not implicate the muscle beneath, but was adherent to the scar. There were no enlarged glands. After removal the tumour measured 5½ by 4 in. Some parts of the growth, which before removal felt quite solid, could be felt to fluctuate from the diminution in tension. On cutting into the tumour, dark-red blood poured out in large quantity. The growth consisted of a number of cysts containing either a very soft white papillomatous growth or altered blood-clot. The blood which flowed away on cutting into the tumour was contained in the cysts. The papillomatous growth was as soft as velvet. It fairly filled the cyst, but was not so tightly packed in as in the primary growth. It was attached to the cyst-wall at one spot, but could be easily washed away by a gentle stream of water. The cyst-wall was in some places fairly thin, in others thick and tough like leather. There was no solid growth between the cysts. The tumour was only adherent to the skin in the way a fatty tumour is. It did not in any way infiltrate

× 100.

*A. Large melanotic cells in stem of papillomatous growth.*
*B.B. Vessels.*

the skin. Sections were made across one of the smaller cysts filled with intracystic growth. With a pocket lens the interior of the cyst was seen to be occupied by an arborescent mass, and papillomatous processes could be seen springing from the wall in parts. Under the microscope these were seen to be formed of a delicate central stem (generally containing a blood-vessel) covered with round cells, the deeper ones having elongated nuclei like columnar cells in parts. The wall of the cyst was in some places fibrous, but in other areas some round and spindle cells were to be seen.

## Figures and Tables

**A f1:**